# Emerging Cellular Therapies for Anti-myeloperoxidase Vasculitis and Other Autoimmune Diseases

**DOI:** 10.3389/fimmu.2021.642127

**Published:** 2021-07-29

**Authors:** Dragana Odobasic, Stephen R. Holdsworth

**Affiliations:** ^1^Centre for Inflammatory Diseases, Department of Medicine, Monash University, Monash Medical Centre, Clayton, VIC, Australia; ^2^Department of Nephrology, Monash Health, Clayton, VIC, Australia; ^3^Department of Immunology, Monash Health, Clayton, VIC, Australia

**Keywords:** vasculitis, glomerulonephritis, myeloperoxidase, tolerogenic dendritic cells, regulatory T cells, stem cells

## Abstract

Anti-myeloperoxidase vasculitis (MPO-AAV) is a life-threatening autoimmune disease which causes severe inflammation of small blood vessels, mainly in the kidney. As for many other autoimmune diseases, current treatments, which consist of general immunosuppressants, are partially effective, toxic and broadly immunosuppressive, causing significant and serious adverse effects in many patients. Therefore, there is an urgent need for more targeted and less harmful therapies. Tolerogenic dendritic cells, regulatory T cells and stem cells have emerged as attractive, new and safer options for the treatment for various autoimmune diseases due to their unique and selective immunosuppressive capacity. In this review, we will discuss how these cellular therapies offer potential to become novel and safer treatments for MPO-AAV.

## Introduction

Anti-neutrophil cytoplasmic antibody (ANCA)-associated vasculitis (AAV) is a severe condition which causes inflammation and damage of small blood vessels. It is caused by autoimmunity against neutrophil proteins, mainly myeloperoxidase (MPO) and proteinase-3 (PR3). AAV consists of microscopic polyangiitis (MPA), granulomatosis with polyangiitis (GPA) and eosinophilic granulomatosis with polyangiitis (EGPA), and it has an annual incidence of 20/million (1, 2). MPO-ANCA are found in the majority of MPA and a smaller proportion of GPA and EGPA patients, while PR3-ANCA are found predominantly in GPA (3).

Although MPO-AAV and PR3-AAV have many similarities, they are now considered to be different diseases based on their epidemiology, genetics, etiology, immunopathology and clinical features. MPO-AAV predominates in southern Europe and Asia-Pacific, is genetically weakly associated with HLA-DQ, mainly occurs as a single event, and vasculitis is mostly limited to the kidney (1, 4). In contrast, PR3-AAV is more commonly found in the northern hemisphere, is associated with HLA-DP, has a higher rate of relapse, and organs other than the kidneys such as the lungs are also affected (1, 4).

As in many other human autoimmune diseases, the aim of conventional therapies in AAV has been to damage the immune system in general to attenuate the autoimmunity-induced inflammatory injury to organs expressing the target autoantigens. The development of biological therapies, mainly monoclonal antibodies, has allowed more accurate selection and targeting of key components of pathways which mediate auto immunopathogenesis. These are more “precise” than conventional therapies in having fewer off target injurious effects on the immune system.

However, the ultimate goal for many autoimmune diseases, including AAV, is to restore tolerance (unresponsiveness) towards the disease-causing autoantigen in a way that would turn off only the injurious autoimmune response, without adversely affecting host immune defense and causing other major side effects. Although we now have tools which can restore tolerance in an antigen-specific manner, antigen-specific immunosuppression causing disease reversal is nearly impossible to achieve in many human autoimmune diseases, but is likely to be realistic in AAV, particularly MPO-AAV, due to the reasons explained below. For a biological treatment strategy to deliver successful antigen-specific restoration of tolerance, there are several essential components:

- The diagnosis of a particular disease must be possible before the immune injury has induced irreversible damage to the target organ. In many human autoimmune diseases such as autoimmune thyroiditis and type 1 diabetes (T1D), this is not possible.
- The major human disease-causing autoantigen needs to be known. This is not the case in many diseases including systemic lupus erythematosus (SLE), rheumatoid arthritis (RA) and type 1 diabetes (T1D).
- In diseases such as Multiple Sclerosis (MS), epitope spreading makes antigen-specific inhibition complex and difficult to achieve.
- There must be thorough and precise knowledge of the key pathways and the essential components of the immune pathogenesis of the disease.
- Animal models with similar autoimmune target, immune pathogenic pathways and clinical outcomes need to exist or can be developed so that pre-clinical studies can be used to provide proof-of-concept that safe precise biological therapies can be taken on to clinical trials.

MPO-AAV is an autoimmune disease where all these criteria are met. Although patients often present with significant target organ (kidney) damage, at the time of diagnosis, the majority have sufficient kidney reserve to live a long life without the need for transplantation or dialysis once effective therapy is introduced. The major disease-causing autoantigen (MPO) is known, there is no evidence of epitope spreading occurring, and relevant animal models have been developed that have helped define the immunopathogenesis of the human disease and identify new therapeutic targets. On the other hand, although humanized mice have emerged as a promising tool to develop successful *in vivo* models of PR3-AAV (5), murine models of this disease have been difficult to induce (6). This is most likely because PR3 is not detected on mouse neutrophils and is therefore not accessible to anti-PR3 antibodies (1). Hence, this review will focus mainly on MPO-AAV. However, the potential cellular therapies discussed below may be also applied to PR3-AAV and later tested in relevant animal models.

**Figure 1:**
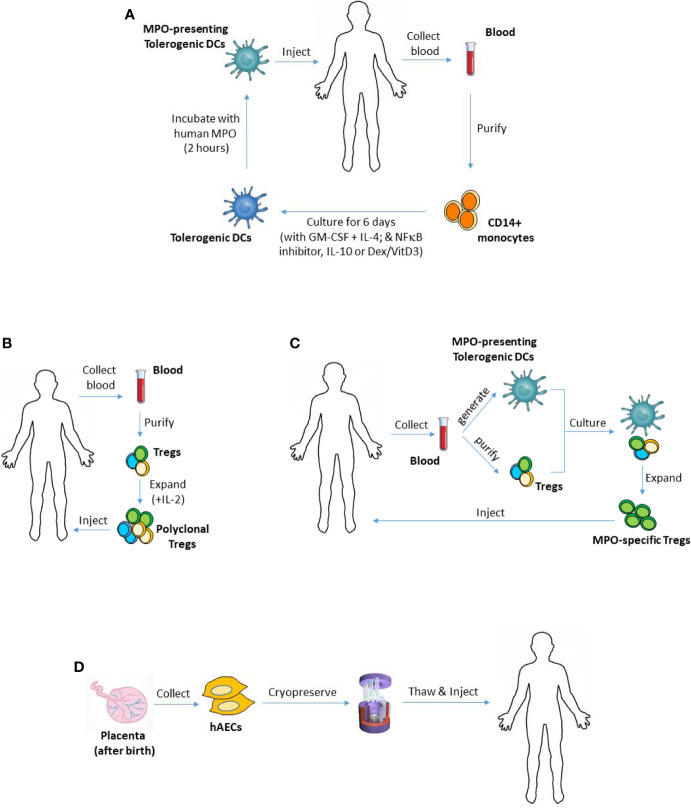
Using tolerogenic DCs, Tregs or hAECs as cellular therapies in MPO-AAV. **(A)** To generate monocyte-derived DCs, CD14+ monocytes can be purified from patient’s blood and cultured in the presence of GM-CSF and IL-4. Different types of tolerogenic DCs can be generated by adding various anti-inflammatory mediators to the culture including IL-10, Dex/VitD3 or inhibitors of NFκB. To make the DCs present MPO, they are then pulsed with purified human MPO, and injected back into the patient. **(B)** To expand polyclonal Tregs *ex vivo*, patient’s Tregs (CD4^+^CD25^+^CD127^-/low^) can be isolated from their blood, then cultured for several weeks in the presence of IL-2. **(C)** Patient’s MPO-presenting tolerogenic DCs and Tregs, generated and purified as in **(A, B)** respectively, could be co-cultured to expand MPO-specific Tregs. Such antigen-specific Tregs are expected to have superior suppressive capacity compared with polyclonal Tregs. **(D)** hAECs are isolated from the amniotic membrane of the placenta after birth and cryopreserved as primary (non-cultured) cells. They can be given to MPO-AAV patients when needed. DCs, dendritic cells; Tregs, regulatory T cells; hAECs, human amniotic epithelial cells; MPO, myeloperoxidase; AAV, ANCA-associated vasculitis; GM-CSF, granulocyte-macrophage colony-stimulating factor; IL-4, interleukin 4; Dex, dexamethasone; VitD3, vitamin D3; NFκB, nuclear factor kappa B.

Autologous *ex vivo*-generated regulatory T cells (Tregs) and autoantigen-loaded tolerogenic dendritic cells (DCs) are an appealing tool for the treatment of autoimmune diseases, including MPO-AAV, because they can provide safe, antigen-specific immunosuppression, without posing any risk of rejection. Alternative to inducing MPO-specific immunosuppression, other attractive cell therapies such as stem cells would desirably suppress anti-MPO autoimmunity and vasculitis without being rejected or producing major adverse effects. This review will discuss emerging evidence which suggests that such cellular therapies may offer a safer, effective therapeutic option for MPO-AAV. **Figure 1** illustrates how these therapies could be used in this disease, with their main advantages and disadvantages summarized in **Table 1**.

**Table 1:** Advantages and disadvantages of different cellular therapies for human MPO-AAV.

Cellular therapy	Advantages	Disadvantages
Antigen-loaded tolerogenic DCs	- antigen-specific - no risk of rejection (using patients’ own *ex vivo*-modified cells) - abundant DCs can be generated from patients’ PBMCs - expensive genetic engineering methods not required	- cost related to cell isolation and *in-vitro* culture - long-term stability and survival after transfer needs to be optimized - migratory capacity can be impaired with certain tolerogenic treatments
Polyclonal Tregs	- expensive genetic engineering methods not required - no risk of rejection (using patients’ own *ex vivo*-expanded cells)	- not antigen-specific - less suppressive than antigen-specific Tregs - reduced stability during *in vitro* expansion due to the loss of foxp3 - cost related to cell isolation and *in vitro* culture
Antigen-specific Tregs	- antigen-specific - no risk of rejection (using patients’ own *ex vivo*-modified cells) - more suppressive than polyclonal Tregs	- some approaches use expensive genetic engineering methods (e.g. specific TCR-Tregs and CAR-Tregs) - difficulty to generate specific TCR-Tregs due to the immunodominant peptide of human MPO being unknown - CAR-Tregs may become systemically hyper-activated due to widespread expression of MPO - cost related to cell isolation and *in vitro* culture
hAECs	- low immunogenicity and risk of rejection - low risk of causing tumors - offer protection against infection, cancer and cardiovascular disease - unique immunosuppressive capacity - isolation from the amnion after birth is non-invasive, easy, fast, relatively cheap and ethical - plentiful cells are isolated from an abundant source (placenta)	- not antigen-specific, thus pose a risk of more broadly suppressing immunity - low long-term survival after administration

## MPO-AAV

MPO-AAV is caused by autoimmunity to MPO, an abundant protein found inside our most common immune cell, neutrophil (3, 7). This disease causes severe inflammation and destruction of small blood vessels, leading to significant morbidity and mortality. It equally affects men and women, mainly over the age of 50, but it can also affect young adults and children (1). MPO-AAV is associated with the presence of serum anti-MPO antibodies, known as anti-neutrophil cytoplasmic antibodies or MPO-ANCA. Although the kidney bares the brunt of autoimmune injury, the major autoantigen, MPO, is not normally expressed in the kidney. MPO is, however, the major protein present in the granules of neutrophils (8). Evidence from relevant human and mouse studies shows that circulating MPO-ANCA target activated, MPO-exposing neutrophils, which subsequently lodge in glomeruli and deposit the autoantigen there (9–16). It also shows that both MPO-ANCA and MPO-specific CD4 T cells vitally contribute to the development of glomerular injury in MPO-AAV (9–16).

Animal models have provided a great deal of knowledge about the immunobiology of MPO-AAV. A few different animal models of MPO-AAV exist, including an antibody transfer mouse model in which injury is mediated by passively-transferred MPO-ANCA, transplanting irradiated, MPO-immunized MPO-deficient mice with wildtype bone marrow and immunizing WKY rats with MPO. They all closely resemble human disease, immunologically and pathologically, and have been thoroughly reviewed elsewhere (17). In one model, wildtype mice are immunized with MPO to induce active MPO-specific autoimmunity, including MPO-ANCA and MPO-specific T cells (16, 18–21). They are then administered either MPO-ANCA or low dose anti-glomerular basement membrane (GBM) globulin which cause neutrophils to deposit MPO in glomeruli for subsequent recognition by infiltrating MPO-specific T cells. In line with data from MPO-AAV patients, CD4 (13, 22) and CD8 T cells (22, 23), neutrophils and macrophages (13, 22, 24), IL-17A (19, 25) and IFNγ (26, 27) promote injury in this model, while CD4+foxp3+ regulatory T cells (Tregs) are inhibitory (28–30). Here, our group has also defined the disease-causing immunodominant MPO T cell peptide (16), which is strikingly similar to the immuno-dominant human MPO peptide (16, 20). Hence, studies of MPO-specific immunomodulation in this model are very relevant to human disease.

As for many other autoimmune diseases, current treatments for MPO-AAV are only partially effective, but are harmful and non-specific, thus causing significant serious side effects in many patients which lead to considerable complications and death. Decades after their introduction, the first-line therapy for induction of remission still consists of high-dose corticosteroids and cyclophosphamide (1). These treatments induce remission in 70-90% of patients, but the incidence of dialysis or death at 5 years is still high (30%) (3, 31, 32). 1 in 3 patients also relapse while being treated (1, 31). The main problem with these therapies is that they are highly toxic and broadly immunosuppressive. Cyclophosphamide increases the risk of infection, cancer and infertility, while corticosteroids cause cardiovascular problems, diabetes, depression, anxiety, insomnia, bone loss and gastric ulcers, as well as an increased risk of infection (32, 33). The rate of infection can be potentially reduced by lowering cumulative doses of corticosteroids or replacing them with avacopan (complement C5a receptor inhibitor) (34, 35). Rituximab (B cell-depleting monoclonal antibody) has been approved for use instead of cyclophosphamide, but it induces similar rates of infections, mainly due to reduced numbers of B cells, hypogammaglobulinemia and late-onset neutropenia (32, 36). Of all patient deaths in AAV, an alarming 70% are caused by treatment-related side effects, mostly infections (31). Another common downside of these therapies, including past and currently-ongoing clinical trials in ANCA vasculitis (1), is that none of them specifically target anti-MPO autoimmunity to avoid complications due to their off-target, non-antigen-specific effects. There is an urgent need for safer, more-targeted effective therapies.

## Tolerogenic DCs

DCs are specialized immune cells and most-potent antigen-presenting cells which vitally control adaptive immunity. After activation, they upregulate MHC-II, costimulatory molecules (e.g. CD40, CD80/86) and pro-inflammatory cytokines (e.g. IL-12, TNF). In this context, they present antigen to T cells *via* MHC-II to induce protective antigen-specific T cell immunity against pathogens. In contrast, DCs are also critical to the maintenance of peripheral tolerance by presenting self-antigens in an immature or semi-mature state, thus causing T cell hyporesponsiveness, as shown by studies in which DC depletion during steady-state resulted in fatal autoimmunity (37, 38). Tolerogenic DCs are found throughout the body including mucosal surfaces where they promote airway and oral tolerance and unresponsiveness towards commensal microbiota (37). They generally express high levels of anti-inflammatory (e.g. PD-L1, IL-10, TGFβ) and low levels of pro-inflammatory mediators (e.g. CD40, CD80/86, IL-12) (37).

Tolerogenic DCs can be also made *ex vivo* by modification with various anti-inflammatory agents including IL-10, Dexamethasone (Dex; glucocorticoid), VitaminD3 (VitD), inhibitors of NFκB (one of their major pro-inflammatory pathways) and tools such as anti-sense oligonucleotides (oligos) which can inhibit gene expression of molecules critical for T cell activation like CD80, CD86 and CD40 (39–46). These DCs use various molecules to inhibit pathogenic T cells (e.g. PD-L1, IL-10, TGFβ), and can turn them off by promoting apoptosis, and by inducing endogenous inhibitory cells such as CD4+foxp3+ Tregs, type 1 regulatory cells (Tr1; inhibitory CD4+foxp3-) and regulatory B cells (Bregs) (39, 40, 47). However, mechanisms of immunosuppression are context and disease-dependent and vary between differently-modified DCs which exhibit different phenotypes.

Various antigen-loaded tolerogenic DCs, including CD40-deficient DCs, DCs treated with Dexamethasone (Dex)/Vitamin D (VitD) or NFκB inhibitors (e.g. BAY-11-7082), have rapidly, in 2-3 days, induced potent antigen-specific immunosuppression and attenuated organ damage in models of allergy, transplantation and autoimmune diseases (e.g. RA, MS, T1D), while generally remaining very stable after transfer (41, 48–51). These DCs have induced long-lasting immunosuppression, although they themselves have persisted for only a few weeks in recipients (52). Due to their stability and capacity to provide antigen-specific immunosuppression, as shown in rodents and non-human primates (41, 48–51), autologous *ex vivo*-derived tolerogenic DCs have emerged as an excellent therapeutic candidate for the treatment of autoimmune diseases. Their clinical use in autoimmunity is now a reality, with 5 phase I trials completed in RA, T1D, MS, neuromyelitis optica (NMO) and Crohn’s disease (53–57). In these clinical studies, patients’ own *ex vivo*-modified tolerogenic DCs (including BAY, Dex/VitD and CD40/80/86 anti-sense oligo-treated DCs), which were biologically active, were shown to be safe and well-tolerated, without producing any major side effects for 12 months. Furthermore, it was shown that tolerogenic DC therapy produced anti-inflammatory and immunomodulatory effects in these trials, with some positive clinical outcomes. For example, in RA, the DCs decreased effector T cells and their ability to produce IL-6, increased the ratio of Tregs: effector T cells, reduced serum levels of pro-inflammatory cytokines and chemokines and diminished the disease activity score (53). In T1D, tolerogenic DCs increased the frequency of peripheral Bregs (54), while in MS/NMO, they upregulated IL-10 production by PBMCs and reduced memory CD8 T cells (57). In Crohn’s disease, tolerogenic DCs reduced the disease activity index, with 1 patient reaching clinical remission and 2 a positive clinical response, and disease lesions markedly improving in 3 out of 9 patients (55). These studies demonstrate the feasibility and safety of this therapy in humans, with promising clinical outcomes which need to be further investigated in future trials. The optimum and most effective treatment regimen still needs to be determined for each autoimmune condition including DC dose, route and frequency of administration, long-term stability and survival, and the type of tolerogenic DC to be used. It also needs to be ensured that these cells can migrate to lymphoid organs to be able to exert their effects since many anti-inflammatory agents used to make tolerogenic DCs can inhibit their migratory capacity.

Recently, we demonstrated that administration of *ex vivo*-generated antigen-presenting tolerogenic DCs can induce selective, MPO-specific immunosuppression and attenuate vasculitis in mice (58). Bone marrow-derived tolerogenic DCs were generated by treatment with an NFκB inhibitor (BAY-11-7082) and pulsed with mouse MPO. These MPO-presenting inhibitory DCs were then given to mice with established anti-MPO autoimmunity. The DCs significantly decreased vasculitis and MPO-specific immunity, including effector CD4 T cell activation, proliferation, survival and pro-inflammatory cytokine production, as well as CD8 T cell and B cell responses. In line with suppressing anti-MPO immunity, MPO/BAY DCs upregulated Treg expression of inhibitory mediators including foxp3, CTLA-4, TNFR2 and IL-10, without affecting Tr1 or Bregs. Studies in Treg-depleted mice showed that the inhibitory effects of MPO/BAY DCs on anti-MPO autoimmunity were dependent on Tregs. Subsequent adoptive transfer/antibody blockade experiments showed that MPO/BAY DC-induced Tregs suppressed anti-MPO immunity and vasculitis *via* IL-10. Further *in vitro* DC : Treg co-culture experiments, supported by *in vivo* antibody blockade studies, showed that MPO/BAY DCs induced IL-10+ Tregs *via* the ICOS/ICOS-ligand pathway.

Importantly, the above-described inhibitory effects of MPO/BAY DCs on anti-MPO immunity were MPO-specific, since the same DCs did not induce Tregs or suppress immunity against an irrelevant antigen. However, in line with augmenting Th2 responses, MPO/BAY DCs did increase circulating IgE levels in recipient mice, indicating that they may adversely exacerbate allergy, but further studies are needed to test that assertion.

Overall, these pre-clinical, proof-of-concept studies demonstrated that MPO-presenting tolerogenic DCs may be a potential MPO-specific therapy for MPO-AAV which deserve further exploration. Future studies will be needed to test other types of MPO-pulsed tolerogenic DCs in pre-clinical models of MPO-AAV because the effectiveness and precise mechanism of immunosuppression varies between different types of tolerogenic DCs which exhibit different phenotypes. This will allow the best DC candidate to be identified for further studies and progression to clinical trials in MPO-AAV. In addition, before such a cellular therapy could be clinically tested, it would be important to determine if patients’ own MPO-presenting tolerogenic DCs could selectively turn off their anti-MPO T cell responses *ex vivo*.

## Tregs

Tregs are a specialized inhibitory subset of CD4+ T cells characterized by expression of CD25 and foxp3, a transcription factor essential for their development, stability and suppressive capacity (59). In humans, including MPO-AAV patients, they are also CD127-low (60, 61). Tregs play a vital role as regulators of pathogenic immunity in various immune-mediated conditions such as allergy, transplantation and inflammation. They are well known to critically maintain peripheral tolerance by inhibiting pathogenic autoreactive T cells and enhancing the tolerogenic capacity of DCs in an antigen-specific manner (62, 63). Tregs can also inhibit immunity and subsequent inflammation leading to organ damage by suppressing other types of injurious immune cells such as neutrophils, macrophages and B cells (62). They provide immunosuppression by expressing various inhibitory mediators including IL-10, TGFβ, IL-35, CTLA-4 and TNFR2 (58, 64–66).

Dysregulation of Treg number and/or function has been associated with the development of several autoimmune diseases including systemic lupus erythematosus, RA, T1D and MS. Similarly, several studies have shown that the frequency and suppressive capacity of Tregs is significantly reduced and negatively correlates with disease in MPO-AAV patients (28, 30). The critical importance of endogenous Tregs as negative regulators of anti-MPO autoimmunity and vasculitis has been confirmed in experimental MPO-AAV. We have shown that Tregs not only inhibit the generation of anti-MPO autoimmunity (29), but that they also suppress established responses of MPO-specific CD4 T cells, CD8 T cells and B cells (58).

Due to their ability to provide antigen-specific immunosuppression, there has been a great deal of interest to develop and use autologous *ex-vivo-*derived Tregs as a potential therapy for autoimmune diseases. Several studies have shown that such Tregs can attenuate pathogenic immunity and thus organ damage in models of transplantation and autoimmunity (67–69). Phase I trials in autoimmune conditions have demonstrated their feasibility and safety in humans (70, 71). However, many optimizations are still required before Tregs can be clinically used such as enhancing their stability and survival post transfer and determining optimum dose and frequency of administration.

*Ex vivo*-derived Tregs, which have been tested as cellular therapies in animal models and human trials, can be broadly categorized as either polyclonal or antigen-specific Tregs.

### Polyclonal Tregs

Many protocols expand Tregs for several weeks with Treg growth factors such as IL-2, without antigen-stimulation (64). This generates polyclonal Tregs of broad specificities. Such Tregs have attenuated immune-mediated organ damage in models of autoimmune conditions and shown relative safety and some efficacy in human trials (e.g. T1D) (70, 71). However, polyclonal Tregs are much less suppressive than their antigen-specific counterparts (72) and they become less stable and suppressive even before administration due to their progressive loss of foxp3 expression during *in vitro* expansion (73). Therefore, although not ideal, autologous *ex vivo*-expanded polyclonal Tregs could be trialled as a treatment in MPO-AAV patients, but their therapeutic efficacy needs to be first tested in pre-clinical models of the disease.

### Antigen-Specific Tregs

Antigen-specific Tregs are a lot more attractive and promising due to their specificity and superior suppressive capacity. They can be generated in different ways to produce Tregs with an antigen-specific T cell receptor (TCR), chimeric antigen receptor (CAR) Tregs or DC-induced Tregs.

#### Antigen-Specific TCR Tregs

Tregs can be transduced in order to express a high-affinity TCR specific for the autoantigen of interest. Such genetically-engineered Tregs have been effective in models of autoimmune diseases such as T1D (68). Some of the biggest challenges in generating autoantigen-specific TCR Tregs for the treatment of autoimmunity have been the lack of knowledge of the disease-causing autoantigen and its immunodominant epitope(s) in most conditions including SLE and RA, as well as antigen and epitope shifting in others such as T1D and MS.

In MPO-AAV, the autoantigen (MPO) is known. Our group has also defined the T cell specific MPO immunodominant epitope in a murine model of the disease, which interestingly shows striking homology with the human MPO dominant peptide recognized by patients’ pathogenic MPO-ANCA (16, 20). Hence, it may be possible to generate murine MPO-specific TCR and test them in pre-clinical models of MPO-AAV in the near future. The human T cell-specific MPO dominant epitope has not yet been identified, however recent studies suggest that there may be several such epitopes present within human MPO (74), thus making the generation of human MPO-specific TCR Tregs for the treatment of MPO-AAV patients more challenging.

#### CAR Tregs

There has also been a great deal of interest in developing CAR Tregs for the treatment of autoimmune conditions. CAR Tregs are genetically-engineered Tregs which contain an extracellular CAR molecule (autoantigen-binding antibody domain), a transmembrane region and an intracellular T cell signaling domain. CAR Tregs are able to migrate to the site of auto-inflammation where they get activated by their specific autoantigen (69). However, for this therapy to be effective, the autoantigen needs to be expressed only at the diseased site. If the autoantigen is also expressed elsewhere in the body, this could cause systemic over-activation of the Tregs, potentially leading to side effects associated with broader immunosuppression. The potential therapeutic efficacy and safety of CAR Tregs in MPO-AAV may be questionable. This is mainly due to the fact that MPO is not only expressed in the target organ (kidney), but that neutrophils, the major source of MPO, and to a lesser extent monocytes/macrophages, also release it into the extracellular space following cell activation in response to bacteria and other pathogens. MPO is also released from neutrophil precursors during its synthesis in the bone-marrow, so this autoantigen is always present in the circulation (8, 75).

#### DC-Induced Tregs

Antigen-specific Tregs can be expanded *ex vivo* from the polyclonal repertoire without genetic engineering by using antigen-presenting DCs. This approach has been largely employed in transplantation due to the knowledge of alloantigens (76, 77). However, it could be also applied in autoimmune conditions in which the autoantigen is known, including MPO-AAV. For example, *ex vivo* DC-induced/expanded Tregs were effective at reducing immunity and organ damage in a model of RA (78). DC-induced Tregs have increased suppressive capacity and stability due to the long-lasting, enhancing effects of DCs on Treg foxp3 expression and stability (76, 78).

We have recently shown that Tregs induced by MPO-presenting tolerogenic DCs can be used to inhibit established anti-MPO immunity and vasculitis in a pre-clinical model of MPO-AAV (58). In these studies, administration of DC-induced CD4+foxp3+ Tregs suppressed MPO-specific autoimmunity including CD4 T cells, CD8 T cells and B cells, and attenuated vasculitis. These proof-of-concept studies suggested that MPO-specific Tregs induced/expanded by MPO-presenting DCs may be a potential and feasible therapy for MPO-AAV. However, the long-term stability and effect of such Tregs needs to be explored in further studies.

Therefore, although many challenges still remain and the therapeutic efficacy of various types of Tregs needs to be tested in pre-clinical models of MPO-AAV, cell therapy utilizing patients’ own *ex vivo*-modified Tregs, particularly antigen-specific ones, remains a promising potential treatment option for vasculitis patients worth further exploring.

## Stem Cells

Stem cells have also emerged as a promising therapeutic approach for the treatment of various inflammatory and autoimmune diseases due to their immunomodulatory ability. Several types of stem cells, including embryonic and mesenchymal stem cells (MSC), have attenuated organ damage in models of immune-mediated diseases and their safety and efficacy have been evaluated in clinical trials (79). One particular stem cell type, human amniotic epithelial cells (hAECs), have gained much attention in recent years as a treatment choice due to their safety and clinical applicability.

hAECs, which have pluripotent stem cell properties (80, 81), represent a novel, safe and affordable therapeutic option for MPO-AAV, for several reasons. They are isolated after birth from the amniotic which is attached to the placenta (82), therefore bypassing ethical barriers that normally occur with other (e.g. embryonic) stem cells. hAEC isolation involves non-invasive, easy, fast and low-cost procedures, resulting in an abundance of readily-available cells which are infused as primary, non-passaged cells (82). In contrast, other types of stem cells (e.g. mesenchymal and embryonic) have ethical issues regarding their isolation or have to be cultured for several weeks to generate enough infusible cells, thus significantly increasing the cost and potential for *in vitro* mal-transformation. hAECs have low immunogenicity because they do not express class IA antigens (HLA-A, HLA-B, HLA-C) or HLA-DR (class II), and are therefore not rejected upon transfer, nor do they form teratomas due to lacking telomerase, as shown in animals and humans (81, 83, 84).

Importantly, consistent with their role to protect the fetus from mother’s immune system, hAECs, like other stem cells, are immunosuppressive. In fact, similar to MSC (85, 86), the protective effects of hAECs are largely due to their paracrine action and immunosuppressive capacity, rather than multilineage differentiation potential. They have suppressed pathogenic immunity by inhibiting effector T cells, altering macrophage polarisation toward the anti-inflammatory M2 phenotype, inhibiting neutrophils (87, 88) or by inducing other immunosuppressive cells such as Tregs and Bregs and as such they have attenuated organ damage in models of various inflammatory and autoimmune diseases including MS, autoimmune thyroiditis and uveitis, lung injury, liver fibrosis (89–94) and stroke (95). Similar inhibitory effects of hAECs on human T cells have been reported *in vitro*. For example, in co-culture experiments with human PBMCs or purified CD4 T cells, hAECs significantly decreased their proliferation and Th1/Th17 cytokine production (96, 97).

hAECs express a range of immunosuppressive mediators, including transforming growth factor beta (TGFβ), prostaglandin-E_2_ (PGE_2_) and the immunosuppressive HLA-G (81, 89), which they use to suppress immunity. However, which anti-inflammatory mediators are utilized and which immune cells are targeted by the hAECs depends on the model used. For example, in bleomycin-induced lung injury, hAECs attenuated disease by inducing Tregs *via* TGFβ (91). In a model of MS, hAECs suppressed lymphocyte proliferation *via* TGFβ and PGE_2_ (89), but in those studies, hAEC-mediated suppression of disease was not associated with Treg induction. This shows that Tregs are required for hAEC-exerted effects in some, but not all, inflammatory models. Interestingly, but similar to MSC, IFNγ/TNF stimulation of hAECs enhances their production of suppressive molecules such as TGFβ (86, 91), suggesting that exposure to pro-inflammatory mediators found in many autoimmune and inflammatory conditions may further augment the inhibitory function of hAECs. Term hAECs are also more suppressive than those from pre-term donors (< 36 weeks gestation), possibly due to their increased expression of HLA-G (98), which is known to suppress T cell and neutrophil responses and induce Tregs (99, 100).

Recently, it was shown that hAECs can also mediate immunosuppression by producing exosomes (88, 101). Exosomes are released microvesicles (~50-100nm in diameter) which contain proteins, lipids and DNA/RNA with important roles in intercellular communication (102). They are released by many cell types and have been successfully used as a cell-free therapy in inflammatory conditions (103, 104). hAEC-derived exosomes inhibit various immune cells including T cells, macrophages and neutrophils *in vitro* and show protection in inflammatory models of acute lung injury and liver fibrosis (88, 101). Therefore, hAEC exosomes may represent a potential cell-derived, but cell-free, therapy in MPO-AAV.

Unlike the current AAV treatments, hAECs are unlikely to cause major side effects because they have anti-infection and anti-cancer properties. They produce various anti-microbial mediators including β-defensins and type I interferons in response to bacteria and viruses *in vitro* (105, 106). They inhibit cancers directly by inducing apoptosis of malignant cells and indirectly by enhancing anti-tumor immunity *in vivo* (107, 108). hAECs also protect against cardiovascular conditions, including stroke and myocardial infarction (95, 109).

hAECs have already entered the clinic. Amniotic membrane or hAECs have been safely used to treat eye injuries and promote wound healing for decades (84, 110). hAECs are also being tested in clinical trials as a therapy for other conditions. A phase I trial in babies with bronchopulmonary dysplasia has demonstrated their short and long-term feasibility and safety (111, 112), while another two phase I trials are underway in liver fibrosis (113) and stroke (114).

Therefore, stem cells such as hAECs represent a potentially safer, effective therapy for MPO-AAV due to their relatively harmless and immunomodulatory profile. Stem cells have never been tested as a treatment in this disease, but we are currently exploring the therapeutic efficacy of hAECs and their exosomes in pre-clinical models of MPO-AAV, which will pave the way for this therapy to be clinically tested in vasculitis patients.

## Conclusions

Overall, although many obstacles still need to be overcome before tolerogenic cell therapy becomes a reality for vasculitis patients, autologous Tregs and *ex vivo*-derived MPO-loaded tolerogenic DCs offer promise to be a feasible and successful antigen-specific treatment for MPO-AAV. This is because (i) Tregs and tolerogenic DCs have been generally shown to be stable, safe and well-tolerated in patients, and they can uniquely induce antigen-specific immunosuppression in various autoimmune conditions including experimental MPO-AAV, and (ii) MPO-AAV itself does not hold any caveats for effective antigen-specific restoration of tolerance. hAECs, if proven to be effective in pre-clinical models of MPO-AAV, may be even closer to clinical testing in MPO-AAV patients since they have already been extensively characterized, provide an off-the-shelf, abundant and relatively cheap therapeutic option, do not get rejected after transfer and clinical trials in other conditions have demonstrated their safety in humans. If successful, these cell therapies have the potential to change clinical practice in MPO-AAV and provide immense benefit to patients by decreasing their risk of death and complications from major adverse and non-antigen-specific effects which currently occur with the existing treatments.

## Author Contributions

DO searched the literature and wrote the manuscript. SH reviewed and edited the paper. All authors contributed to the article and approved the submitted version.

## Funding

The funds for these studies were used from grants provided by the National Health and Medical Research Council of Australia (NHMRC) and the Australian Government Department of Health [Medical Research Future Fund [MRFF)].

## Conflict of Interest

The authors declare that the research was conducted in the absence of any commercial or financial relationships that could be construed as a potential conflict of interest.

## Publisher’s Note

All claims expressed in this article are solely those of the authors and do not necessarily represent those of their affiliated organizations, or those of the publisher, the editors and the reviewers. Any product that may be evaluated in this article, or claim that may be made by its manufacturer, is not guaranteed or endorsed by the publisher.
